# First Identification of a Large Set of Serine Hydrolases by Activity-Based Protein Profiling in Dibutyl Phthalate-Exposed Zebrafish Larvae

**DOI:** 10.3390/ijms232416060

**Published:** 2022-12-16

**Authors:** Rodrigue S. Yedji, Bénédicte Sohm, Virginie Salnot, François Guillonneau, Carole Cossu-Leguille, Eric Battaglia

**Affiliations:** 1 LIEC, UMR7360, Campus Bridoux, Université de Lorraine, 57070 Metz, France; 2 Plateforme Protéomique 3P5, Inserm U1016-Institut Cochin, MICUSPC, Université Paris Descartes, 75006 Paris, France; 3 Unité Protéomique Clinique, Institut de Cancérologie de l’Ouest, CRCI2NA-UMR INSERM 1307/CNRS 6075, team03, 15, rue André Boquel, 49055 Angers, France

**Keywords:** zebrafish larvae, serine hydrolases, dibutyl phthalate, activity-based protein profiling, proteomics

## Abstract

Despite the involvement of several serine hydrolases (SHs) in the metabolism of xenobiotics such as dibutyl phthalate (DBP), no study has focused on mapping this enzyme class in zebrafish, a model organism frequently used in ecotoxicology. Here, we survey and identify active SHs in zebrafish larvae and search for biological markers of SH type after exposure to DBP. Zebrafish were exposed to 0, 5, and 100 µg/L DBP from 4 to 120 h post-fertilization. A significant decrease in vitellogenin expression level of about 2-fold compared to the control was found in larvae exposed to 100 µg/L DBP for 120 h. The first comprehensive profiling of active SHs in zebrafish proteome was achieved with an activity-based protein profiling (ABPP) approach. Among 49 SHs identified with high confidence, one was the carboxypeptidase ctsa overexpressed in larvae exposed to 100 µg/L DBP for 120 h. To the best of our knowledge, this is the first time that a carboxypeptidase has been identified as deregulated following exposure to DBP. The overall results indicate that targeted proteomics approaches, such as ABPP, can, therefore, be an asset for understanding the mechanism of action related to xenobiotics in ecotoxicology.

## 1. Introduction

Dibutyl phthalate (DBP) is a plasticizer belonging to the phthalate ester family widely found in various environmental compartments [1,2]. In general, phthalate esters enter the environment through losses during manufacturing processes via air emissions, aqueous effluents, solid wastes and leaching of end products, since they are not chemically bound to the polymer matrix [2,3]. Several phthalates, including DBP have been classified by the European Chemicals Agency (ECHA) as endocrine disruptors [4]. They have numerous effects on living organisms, such as malformations, anti-androgenic activity, teratogenicity, peroxisome proliferation and reproductive toxicity in laboratory animals [2]. These effects involve several receptors, including those activated by peroxisome proliferators (PPARs) [5,6], aryl hydrocarbon receptors (AhR) [7], estrogen receptors (ERs) [8,9] and nuclear thyroid hormone receptors (TRs) [9,10,11]. Although the toxic effects of phthalates are well known, their modes of action are not fully established.

In this context, the study of enzymes involved in phthalate metabolism, as well as those whose expression is sensitive to the exposure to phthalates may be a relevant area to explore. Enzymes such as carboxylesterases (CES), acetylcholine esterase (AChE) or butyrylcholine esterase (BchE) all belonging to the family of serine hydrolases (SHs) are reported for their role in the metabolism of xenobiotics, including phthalates [12,13]. Carboxylesterase is also used as a biomarker of xenobiotic exposure in zebrafish [14,15]. Likewise, Acey et al., (2002) demonstrated in brine shrimp (*Artemia salina*) larvae that there was a correlation between acute DBP toxicity and the expression of BChE, a hydrolyzing enzyme of this phthalate ester [16]. In addition to BChE, AChE, a sensitive biomarker of neurotoxicants, is frequently used to assess phthalate neurotoxicity [17,18]. Xu et al. (2013) reported the inhibition of AChE in zebrafish larvae exposed to DBP and diethyl phthalate [19]. Despite the involvement of several SHs in the metabolism of xenobiotics and phthalates in particular and the sensitivity of their expression or activity to these compounds, no study has focused on the overall mapping of this enzyme class in zebrafish (*D. rerio*).

SHs are one of the largest and most diverse enzyme families in eukaryotic and prokaryotic proteomes [20,21,22,23]. They share a base-activated serine nucleophile catalytic mechanism to hydrolyze amide, ester and thioester bonds in small-molecule metabolites, peptide and protein substrates [24,25]. They play important roles in numerous developmental and tissue-specific events in vivo, including peptide hormone processing and neural plasticity [26]. A thorough evaluation of the SHs change in expression upon exposure to phthalates could help shed light on their modes of action. This assessment can be performed using a chemoproteomic approach called activity-based protein profiling (ABPP), which uses a reactive probe to serine hydrolases. This probe allows the labelling and subsequent identification of enzymes through the chemical recognition of a specific catalytic mechanism [27].

The present study surveys the global activity of SHs in 5-day post-fertilization (dpf) zebrafish larvae. In order to validate the activation of molecular pathways sensitive to DBP in exposed organisms, we associated with ABPP the measurement of known biomarkers by two classic approaches, i.e., gene expression by RT-qPCR (vitellogenin, vgt; 17β-hydroxysteroid dehydrogenase, 17β-hsd and Acyl-coenzyme-A-oxidase, acox-1) and the enzymatic activity of AChE. In addition, we monitored survival and developmental abnormalities in control and DBP-exposed larvae. The comparison of SHs activity profile between exposed organism and control was assessed in order to gain insight into larvae response to DBP exposure.

## 2. Results

### 2.1. Survival and Developmental Abnormalities in Control and DBP-Exposed Embryos and Larvae

DBP concentrations were chosen to mimic low (5 µg/L, C5) and high (100 µg/L, C100) environmental contaminations. Mortality was recorded only in the first 48 h of exposure. It did not differ significantly (*t*-test, *p* < 0.05) between DBP-exposed and unexposed embryos. Mortality averaged 2% per treatment (Table S1). Exposure to DBP did not result in developmental abnormalities. Overall, hatching rates were 1.3%, 69% (± 6%) and 96% (± 1%) at 72 hpf, 96 hpf and 120 hpf, respectively without a significant difference (*t*-test, *p* < 0.05) between control and treatments (Table S1). Overall, C5 and C100 concentrations are, thus, environmentally relevant and without obvious toxicity (Table S1). Furthermore, we also evaluated a high (500 µg/L) DBP concentration but this concentration led to high mortality and developmental abnormalities and was, therefore, not further tested.

### 2.2. AChE Activity Assay

**Figure 1:**
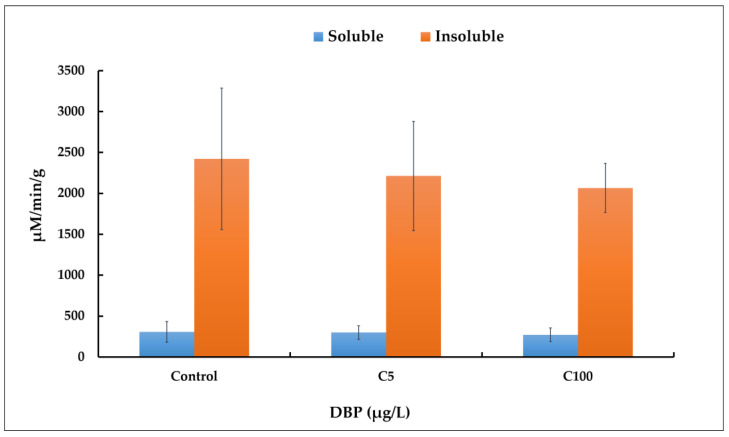
AChE activity in zebrafish (*D. rerio*) larvae exposed to different concentrations of DBP (0 or control, 5 and 100 µg/L noted, respectively C5 and C100) for 5 days. Measurements were performed on the soluble and insoluble protein fractions. Results are the mean ± SD of replicate samples. For controls, AChE activity was 307 µM/min/g (±127) and 2422 µM/min/g (±863) for soluble and insoluble fractions, respectively. A *t*-test was used to determine statistical differences between experimental groups, with *p* < 0.05 considered as significant.

The reduction of AchE activity is often used as a biomarker of neurotoxicity in fish species, including zebrafish [19]. As this enzyme can exist in a molecular form that is both membrane-bound and secreted into cells [28], activities were measured on both soluble and insoluble protein fractions. The average AChE activity is seven times higher in the insoluble fractions compared to the soluble ones (Figure 1). No statistically significant reduction of AChE activity was observed in soluble nor insoluble fractions during DBP exposure (*t*-test, *p* < 0.05).

### 2.3. qRT-PCR Expression of Relevant Genes

**Figure 2:**
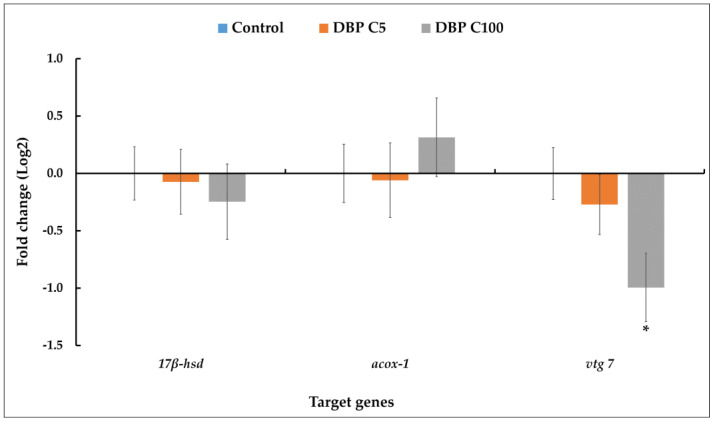
Disruption of the expression of three target genes (*acox1, 17β-hsd, vtg 7*) revealed by qRT-PCR in zebrafish larvae exposed to solvent (DMSO control) and DBP (5 µg/L, C5 and 100 µg/L, C100) for 5 days. The CT values of each gene of interest were normalized to the expression of the housekeeping genes (rpl13a and EF1a) by using the 2^−ΔΔCt^ [29]. Results are given as mean ± standard deviation (n = 4) for each treatment group. A *t*-test was used to determine statistical differences between experimental groups and *p* < 0.01 was considered as significant. The asterisk shows a statistically significant difference (*p* < 0.01) between control and exposed larvae.

The expression of three relevant genes was measured by qRT-PCR to check whether the associated molecular pathways are affected by DBP. For C100 (100 µg DBP/L), *vtg* expression level (Figure 2) was significantly halved compared to the control. Although the differences were not statistically significant (*p* < 0.01), there was a downward trend in the relative level of *17β-hsd* mRNA and an upward trend in the relative level of *acox-1* mRNA in larvae exposed to C100 compared to control. No change in the relative expression level of the three genes was observed in larvae exposed to C5 (5 µg DBP/L) compared to control.

### 2.4. Validation of the Use of ABPP Probes for Profiling SHs in the Zebrafish Larvae Proteome

**Figure 3:**
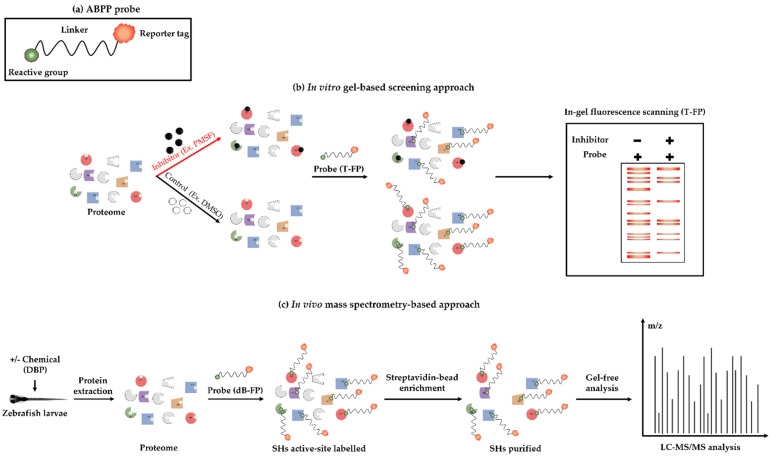
Schematic representation of ABPP approach used. (**a**) Generic structure of an activity-based probe having three components, i.e., the reactive group, a linker, and a reporter tag. (**b**) Protein active-site labelling by competitive ABPP assay (in vitro). For this, zebrafish proteome was pre-incubated with either inhibitor (PMSF) or DMSO, followed by labelling with TAMRA-FP (T-FP). Proteins were separated by SDS-PAGE, followed by in-gel fluorescent scanning to detect the labelled SHs. (**c**) Protein active-site labelling by ABPP assay (in vivo). Zebrafish embryos were exposed to DBP (0, 5 and 100 µg/L) for 120 h. After exposure, larvae were collected and proteome were extracted, then, SHs were labelled with desthiobiotine-FP (dB-FP) probe followed by streptavidin enrichment before being analyzed by gel-free LC-MS/MS.

An in vitro ABPP competition using an activity-based probe was performed together with a known SHs inhibitor, Phenylmethylsulfonyl fluoride (PMSF), to verify the specificity and selectivity of the identified SHs on gel (Figure 3a,b). The potential labelling of the dB-FP probe expected for affinity purification of SHs in zebrafish proteome (Figure 3c) was also tested.

**Figure 4:**
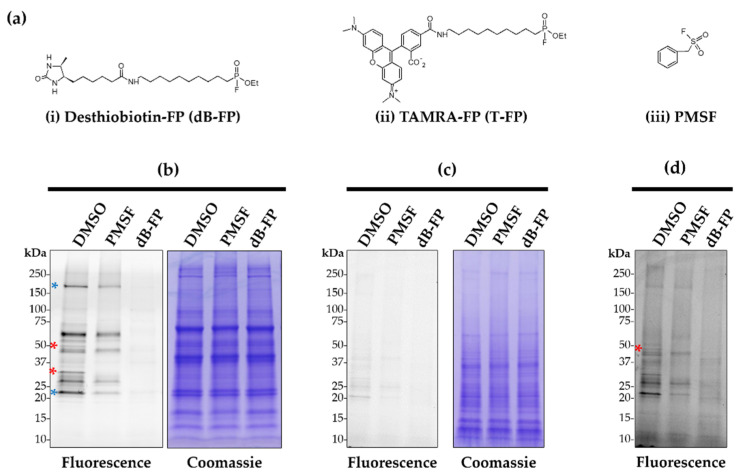
On-gel fluorescence labelling of zebrafish serine hydrolases. (**a**) Structures of the FP probes (T-FP and db-FP) and serine hydrolase inhibitor (PMSF) used. (**b**) One-dimensional gel visualization of the soluble protein fraction of labelled serine hydrolases. In vitro competitive profiling was performed by incubating the proteins at 25 °C for 15 min with the competitor or DMSO (control without competitor), followed by labelling with the T-FP probe for 15 min. The competition was performed with 2 mM PMSF and 2 µM dB-FP. After the fluorescence scan, the gel was stained with Coomassie blue to visualize the protein profile (right picture). (**c**) Competitive profiling of the membrane fraction as in (**b**). (**d**) Shows the high-contrast fluorescence scan of the insoluble fraction performed with ImageJ. A blue asterisk indicates an example of a protein whose labelling is partially inhibited by PMSF, while a red asterisk shows a protein whose labelling is totally inhibited (Figure 4b,d).

For these in vitro competition assays, fractions were pre-incubated with dB-FP, PMSF or DMSO before labelling with the T-FP probe. Tagged proteins were detected in both fractions, with stronger signals in the soluble one (Figure 4b vs. Figure 4c, lane 1). The profiles of labelled proteins differ from one fraction to another (Figure 4b,c) and support our choice to analyze them separately in the proteomic analyses described below. Pre-incubation of the fractions with PMSF totally inhibited the labelling of many proteins in soluble fraction (Figure 4b, e.g., red asterisk) and reduced the labelling of others (Figure 4b, e.g., blue asterisk). These results suggest that these proteins are SHs. Despite the weak labelling in insoluble fraction, several proteins could be detected (Figure 4d, red asterisk), whose signal was partially or totally inhibited by PMSF. However, such imaging will not reveal weakly expressed SHs. These data support the utilization of these two probes for gel imaging and MS identification of larvae SHs.

### 2.5. Identification of SHs in the Proteome of Zebrafish Larvae Exposed to DBP

Because of its sensitivity for protein target identification, gel-free MS-based ABPP was used as described in Figure 3c. The SHs identified in both fractions in control and DBP-exposed larvae (C5 & C100) are listed in Table 1. In total, 50 serine hydrolases were identified in C100 and control, and 53 in C5 samples. Among the 49 SHs common to all three conditions, six are enriched in both fractions, 42 are specifically enriched in soluble fraction and only one SH (tripeptidyl-peptidase 2) is specifically enriched in insoluble fraction. Among proteins other than the SHs identified (Table 1), the collagen-like tail subunit (single strand of homotrimer) of asymmetric acetylcholinesterase was specifically enriched in the insoluble fraction. This protein is mainly responsible for AChE presence at neuromuscular junctions [30].

### 2.6. Functional Annotation of SHs

We focused on SHs comparison between larvae exposed to DBP and control. Only one significantly differentially regulated SH (*p* < 0.05) was observed (Table S2). This enzyme is the carboxypeptidase ctsa, identified in the soluble and insoluble fractions but for which the *t*-test is only significant for the insoluble fraction. It is 2.3 times more expressed at C100 compared to control. In order to obtain additional information on the identified proteins, we performed an analysis with the bioinformatics resources of the STRING database (https://string-db.org/ (accessed on 25 March 2022)) [31]. As for gene ontology (GO) classification of biological process, all annotated proteins are associated with metabolic process (Table S3). About 10% of proteins are so far not annotated in GO biological process. Concerning molecular function, 96% of identified proteins are reported to have a catalytic activity. Among proteins associated with KEGG pathways, we found ctsa differentially regulated according to exposure to DBP. It is involved in the KEGG pathway of lysosomes (KEGG ID dre04142). Only nine proteins were associated with KEGG pathways (Table S3).

We performed a BLASTP search of all 49 SHs against human reference databases (RefSeq, as *D. rerio* sequences are not included) in order to analyze them with the QIAGEN IPA software. A total of 37 SHs were mapped on canonical pathways with the most contributing SHs related to triacylglycerol degradation, retinol biosynthesis, heparan sulfate biosynthesis (late stages), xenobiotic metabolism PXR signaling pathway, SPRINK1 pancreatic cancer pathway, phospholipases and stearate biosynthesis (animals). Of the 37 human orthologous SHs identified, four are used as biomarkers, i.e., FASN for the diagnosis of breast cancer, PLA2G7 for the diagnosis of ischaemic stroke and coronary artery disease, LCAT for the treatment efficacy of dyslipidaemia and CELA1 for the treatment efficacy of pancreatic cancer.

## 3. Discussion

Although there is evidence of involvement of certain SHs in the metabolism of phthalates, including DBP in various organisms [13,16,19], no studies have investigated SHs in zebrafish. We proposed to fill this gap by using ABPP to map active SHs in zebrafish larvae proteome and test the hypothesis that disruption of the activity profile of some SHs could be associated with exposure to DBP. Zebrafish was chosen because of the many advantages attributed to its use [32,33,34,35], as well as for its sensibility to DBP [36,37]. DBP-sensitive biomarkers were also tested to ensure that different metabolic pathways are likely to be affected in zebrafish larvae under our experimental conditions. These include the perturbation AChE activity [19], as well as the transcription level of three target genes (*vtg*, *17β-hsd*, *acox-1*) that are often deregulated in the presence of DBP [38,39,40].

We profiled AChE activity and observed no significant changes between the three conditions of exposure (Figure 1). This finding suggests that there is no major disturbance of neuronal development in zebrafish larvae exposed to DBP (5 and 100 µg/L) based upon AChE, which is in line with Xu et al. (2013), where no significant inhibition of AChE was found after exposure of zebrafish larvae to 5 and 50 µg/L DBP. In contrast, exposure of zebrafish larvae to high doses of DBP (500 µg/L) leads to a significant reduction of AChE activity [19]. We hypothesize that the tested DBP concentrations are not high enough to affect metabolic pathways related to the regulation of AChE activity, particularly those involving AhR [7,41]. The high activity of AChE observed in insoluble fraction could be linked to the abundance of ColQ protein with which AChE subunits would be associated [30].

We then carried out qRT-PCR analyses to assess *acox-1*, *17β-hsd* and *vtg* gene relative expression. The peroxisomal enzyme acox-1 is widely used as an indicator of PPAR activation in fish [39]. No significant variations in relative *acox-1* mRNA levels were observed between the three conditions in this study (Figure 2). Conversely, Uren-Webster et al. (2010) demonstrated that exposure of adult male zebrafish to 5000 mg/kg of the related phthalate DEHP increases the expression levels of two PPAR-responsive genes, including *acox1* [42]. The lack of variation in *acox-1* expression may, therefore, be linked to our experimental conditions (age of the organisms, phthalate concentration and duration of exposure), but also to the fact that DBP is a weaker peroxisome proliferator than DEHP [43]. Gene *17β-hsd* has the ability to modulate not only steroid but also fatty acid and bile concentrations [44], and is considered a key regulator of early embryonic development by allowing maintenance of steroid homeostasis, regulation of cell differentiation and organogenesis [45]. Similar to *acox-1*, no significant change in relative expression of *17β-hsd* was observed in the present study, although there appeared to be a trend towards a reduction at C100.

Regarding *vtg*, a 2-fold reduction in its expression level compared to control was observed in larvae at C100. Many studies suggest the induction of vitellogenin as a marker of toxicity to endocrine-disrupting chemicals (EDCs), but some chemicals appear to influence fish vitellogenin expression in a way that differs from the known pattern [46]. For example, when zebrafish were exposed to 100 µg/L DBP, *vtg* expression was inhibited at 6 dpf but increased at 21 dpf and was not modified at 35 dpf [38]. This variation in *vtg* expression reflects the complexity of the changes induced by EDCs in fish biomarkers, particularly in zebrafish.

Referring to the activity of AChE and genes’ expression level in the presence of DBP, we can assume that the corresponding DBP-sensitive molecular pathways are not fully affected under our experimental conditions. On other hand, vtg mRNA disruption suggests otherwise. This indicates that corresponding pathways are differentially activated by DBP.

An ABPP approach was used for the first time in zebrafish larvae to map SHs and evaluate their profiles as a function of exposure to DBP. Classically, SHs are divided into two subgroups consisting of serine proteases (SPs) and metabolic serine hydrolases (mSHs) [21]. We identified, with high confidence, 16 serine proteases (33%) and 33 metabolic serine hydrolases (67%) in the larval zebrafish proteome (Table 1). In addition, we detected a carboxypeptidase (ctsa) overexpressed in the presence of 100 µg/L of DBP. To the best of our knowledge, this is the first time that a carboxypeptidase has been identified as deregulated following exposure to DBP. For example, this protein is not one of the 41 proteins deregulated in the presence of DBP, identified in zebrafish with the untargeted differential proteomics iTRAQ method [47]. Targeted proteomics approaches such as ABPP can, therefore, be an asset for understanding the mechanism of action related to xenobiotics in ecotoxicology.

However, the strong variabilities between biological replicates failed to highlight with confidence more SHs affected by the presence of DBP. It is well established that the embryonic membrane and chorion form a protective barrier and, thus, reduce the sensitivity of zebrafish embryos to chemicals [15,48,49]. At 96 hpf, 31% of the embryos were still protected by the chorion, while the absence of chorion in 69% of the larvae made them more likely to be susceptible to DBP exposure. This biological variation between individuals in the same group may have prevented the observation of differences in the expression of SHs, especially as standard deviations measured between replicates in the same group are high as showed in AChE activity (Figure 1). This potential pitfall has already been reported [50].

About 70% of human genes have at least one obvious zebrafish orthologue [51]. Identification, in this study, of four SHs human orthologue used as biomarkers in the diagnosis and treatment efficacy of several forms of cancer (PLA2G7, CELA1 and FASN) [52,53,54], dyslipidemia (LCAT) [55] and ischemic stroke (PLA2G7) [56], suggests that zebrafish (larvae) may be a relevant model for the SHs inhibitor discovery process (e.g., drug discovery [35]).

The present study demonstrates the utility of the ABPP approach to map active SHs in zebrafish larvae. Functional annotation of 49 SHs common to all three conditions (control, C5, C100) provides new information on the involvement of these enzymes in various metabolic pathways. We identify the carboxypeptidase ctsa as a SH induced by DBP at C100. Further work using different zebrafish developmental stages, DBP concentrations and orthogonal assays, such as Western Blot, against ctsa will help to validate this protein as a potential biomarker for DBP exposure. This will require the production of a zebrafish ctsa antibody. The variability in biological replicates limits the identification of other dysregulated SHs and highlights the importance of a high number of replicates in hatching embryos. Considering the use of xenobiotic-metabolizing enzymes as biomarkers [57,58,59], it seems appropriate to continue to assess SHs expression in relation to DBP or other xenobiotic exposure in order to discover sensitive biomarkers using this approach.

## 4. Materials and Methods

Chemicals and reagents. Dibutyl phthalate (catalog #524980), dimethyl sulfoxide (catalog #D4540), phosphate-buffered saline (catalog #P4417), tris(2-carboxyethyl) phosphine hydrochloride (catalog #75259) and phenylmethanesulfonyl fluoride (catalog #P7626) were purchased from Sigma Aldrich (Darmstadt, Germany). ActivX Desthiobiotin-FP serine hydrolase probe (catalog #88317), ActivX TAMRA-FP serine hydrolase probe (catalog #88318), HaltTM phosphatase inhibitor cocktail (catalog #78420), Dynabeads^TM^ MyOne^TM^ Streptavidine C1 (catalog #65001) and Pierce^TM^ Rapid Gold BCA Protein Assay kit (catalog #A53225) were purchased from Thermo Fisher Scientific (Waltham, MA, USA), and acetone (catalog #412502) was from Carlo Erba reagents (Val de Reuil, France).

Animals and exposure. The zebrafish used were wild-type (AB strain). They were kept in standardized breeding systems supplied by a continuous flow of water in accordance with standard NF EN ISO 7346-2 (1998). The preparation and collection of zebrafish embryos were performed according to Mu et al. (2013) [60]. We performed two series of exposures of two replicates each. Two concentrations of DBP, 5 and 100 µg/L (noted C5 and C100), were tested against a control. All exposure solutions, including the control solvent contained the same concentration of DMSO (0.005% *v*/*v*). For each of the two series of exposure, the embryos used were collected from two independent mating pairs. Two hundred embryos (4 hpf) were exposed in 250 mL at 26 °C with a photoperiod of 14 h/10 h (light/dark) up to 120 hpf. According to the EU Directive 2010/63/EU, there is, therefore, no need for ethical committee approval (only independently feeding larval stages are protected, which is not the case in our work). Every 24 hpf, 80% of the exposure media were renewed with a freshly prepared medium. Observations were made daily to identify abnormalities in growth and egg division, as well as mortalities; those embryos were systematically removed. Twenty-five larvae were then randomly collected for RT-qPCR analyses and the remainder for the proteomic analyses. Samples were stored at −80 °C.

Preparation of protein fractions. All steps were performed at 4 °C. The lysis buffer was composed of 25 mM Tris-HCl pH 7.4, 150 mM NaCl, 1% (*v*/*v*) NP-40, 1 mM EDTA and 5% (*v*/*v*) glycerol and phosphatase inhibitor (PI). Zebrafish larvae were thawed at 4 °C in ice. The larvae were quickly Dounce-homogenized in the presence of 50 µL of lysis buffer and a scoop of glass beads and then 450 μL of lysis buffer were added and a second homogenization was performed with a bead-beater (2 times, 60 s at 30 Hz). The sample was centrifuged at 1500× *g* for 10 min and the supernatant was ultracentrifuged at 100,000× *g* for 60 min to obtain the soluble (supernatant) and membrane (pellet) fractions.

Measurement of AChE enzymatic activities. AChE activity was measured according to the method of Ellman et al. (1961) [61], adapted for measurement in a 96-well plate. The reaction medium consists of 180 µL of 5,5′-Dithiobis(2-nitrobenzoic acid, DTNB) solution (0.355 mM, 0.1 M phosphate buffer pH 7.5), 10 µL of acetylcholine iodide (9.54 mM) and 10 µL of protein sample. The reaction rate was measured in triplicate for each sample at 405 nm for 3 min (one measurement every 30 s). The relative enzymatic activity was calculated considering the control sample (DMSO) as a reference.

Total RNA extraction and reverse transcription. Total RNAs from zebrafish larvae were extracted with the RNeasy kit ((Qiagen, Hilden, Germany) according to the supplier’s instructions. RNA purity and quantity were assessed by spectrophotometry (260 nm and ratio 260/280 and 260/230) and RNA integrity was checked using Bioanalyser 2100 (Agilent, CA, USA). Reverse transcription (RT) was performed with 1 μg of total RNA using SuperScript^®^ IV Reverse Transcriptase from Invitrogen (Life Science). The generated cDNAs were stored at −20 °C until use. The choice of target genes was based on data from the literature. In total, 2 household genes and 3 genes of interest were chosen. The genes of interest were those whose expression was reported to be altered in the presence of a phthalate-type EDC. These are vitellogenin (*vtg*) [62,63], 17β-hydroxysteroid dehydrogenase (*17 b-hsd*) [63] and Acyl-coenzyme-A-oxidase (acox-1) [42]. For the standardization of the results, 2 household genes were selected: ribosomal protein L13a (*rpl13a*) and elongation factor 1α (*ef1α*) [64]. The specific primer sequences (Table 2) were extracted from the literature for some genes, while for others the primers were designed with the bioinformatics tools Beacon Designer™ (Premier Biosoft) and Primer3Plus. The PCR efficiency tests were carried out on the pairs of primers (2.5 µM) before use, and were between 94% and 105%. Hybridization/amplification were performed at 60 °C for 1 min × 40 cycles. The CT values of each gene of interest were normalized against the expression of the housekeeping genes rpl13a and ef1α (geometric means of CT). Relative quantification was performed using the 2^−ΔΔCt^ method [29] and standard deviation was calculated between four biological replicates.

Competitive labelling with T-FP. Competitive labelling was carried out first between a known inhibitor of serine hydrolases, PMSF (2 mM in DMSO) and the ActivX-TAMRA-FP probe (T-FP), and secondly, between the ActivX Desthiobiotin-probe FP (dB-FP) and the T-FP probe, each at 2 µM in DMSO. A total of 50 µg of each fraction (membrane and soluble) were incubated with the inhibitor or DMSO for 15 min at 25 °C, then 2 μM of T-FP probe were added, and samples were incubated again for 15 min. The reaction was quenched by adding SDS-PAGE Laemmli buffer and samples were run on SDS-PAGE. Visualization of enzyme profiles was performed by scanning the gel at 532 nm with the Typhoon™ Imager (GE Healthcare, Chicago, IL, USA). Gels were then stained with Coomassie blue and scanned.

Labelling with dB-FP and affinity purification. The ActivX Desthiobiotin-FP (dB-FP) probe was used for labelling and affinity purification of zebrafish proteome (from soluble and insoluble fractions). For each affinity purification with dB-FP, the same starting protein amount was used, i.e., 650 µg for the soluble fraction and 150 µg for the insoluble fraction due to the small number of organisms used for the exposure experiments. Briefly, the proteins were incubated with 2 µM dB-FP (in 2% DMSO *v*/*v*) or 2% DMSO (no-probe control) at 25 °C for 60 min with occasional gentle mixing. The proteins were then denatured with 0.5% *w*/*v* SDS at 95 °C for 5 min, then allowed to cool down to room temperature before being precipitated with cold acetone to remove excess probe. Precipitation was performed by adding 4 volumes of cold acetone to each sample and then incubated at −20 °C for 60 min. The samples were then centrifuged at maximum speed (21,000× *g*) for 10 min at 4 °C. Pellets were taken up with PBS containing 0.05% *w*/*v* SDS, 1 mL for the soluble fraction and 300 μL for the insoluble (membrane) fraction. Streptavidin beads (DynabeadsTM MyOneTM Streptavidin C1) were washed (3 times with PBS, 150 mM NaCl) and then added to the soluble (50 µL) and insoluble (10 µL) denatured samples. They were then incubated overnight at 4 °C and 6 rpm on an orbital shaker (Hula-Mixer, Invitrogen, Waltham, MA, USA)). The paramagnetic beads were washed twice with the washing solution (PBS, 150 mM NaCl) and one last time with PBS. Protein targets attached to the beads were eluted by heating the beads at 95 °C for 5 min with an eluting solution (50 mM Tris-HCl pH 8.5, 2% *w*/*v* SDS and 10 mM TCEP). The eluted enzyme targets were stored at −20 °C until analysis by mass spectrometry at the Proteom’IC core facility.

### Mass Spectrometry

LC-MS/MS sample preparation. The eluted proteins were digested by “S-Trap” Micro Spin Column (from ProtiFi, Huntington, NY, USA) using 1 µg trypsin (sequencing-grade from Promega, Madison, WI, USA), mostly according to manufacturer’s instruction with modifications: the totality of the sample was denatured *v*/*v* with a solution containing 4% *w*/*v* SDS (GE Healthcare), 400 mM TEAB (Sigma Aldrich), 20 mM TCEP (Sigma Aldrich) and 100 mM Chloro-acetamide (Fluka, Darmstadt, Germany) for 5 min at 95 °C. The samples were acidified with 12% phosphoric acid at 1:10 (acid: protein volume ratio). A total of 6 volumes of S-Trap Binding Buffer (90% *v*/*v* Methanol, 100 mM TEAB) were added. The resulting protein suspension was transferred to the S-Trap filter. After centrifugation and wash with the same buffer, the filter was incubated with 1 µg of Trypsin in 100 µL of 50 mM TEAB at 37 °C overnight. Resulting peptides were dried by speed-vacuum (speed-vac from Eppendorf, Hambourg, Germany), then solubilized in 10 μL of 0.1% *v*/*v* TFA containing 10% *v*/*v* acetonitrile (ACN).

Liquid Chromatography-coupled Mass spectrometry analysis (LC-MS). Analyses were performed on a Dionex U3000 RSLC nano-LC- system (Thermo Fisher scientific) coupled to a TIMS-TOF Pro mass spectrometer (Bruker Daltonik GmbH, Bremen, Germany). One μL was loaded, concentrated and washed for 3 min on a C_18_ reverse phase precolumn (3 μm particle size, 100 Å pore size, 75 μm inner diameter, 2 cm length, from Thermo Fisher Scientific). Peptides were separated on an Aurora C_18_ reverse phase resin (1.6 μm particle size, 100 Å pore size, 75 μm inner diameter, 25 cm length) mounted to the Captive nanoSpray Ionisation module, (IonOpticks, Middle Camberwell Australia) in a 60 min run time with a gradient ranging from 99% of solvent A containing (*v*/*v*) 0.1% formic acid in milliQ-grade H_2_O to 40% of solvent B containing 80% acetonitrile, 0.085% formic acid in mQH_2_O. The mass spectrometer acquired data throughout the elution process and operated in DDA PASEF mode with a 1.1 s/cycle, with timed ion mobility spectrometry (TIMS) mode enabled and a data-dependent scheme with full MS scans in PASEF mode. This enabled a recurrent loop analysis of a maximum of the 120 most intense nLC-eluting peptides, which were CID-fragmented between each full scan every 1.1 s. Ion accumulation and ramp time in the dual TIMS analyzer were set to 50 ms each and the ion mobility range was set from 1/K0 = 0.6 Vs⋅cm^−2^ to 1.6 Vs⋅cm^−2^. Precursor ions for MS/MS analysis were isolated in positive mode with the PASEF mode set to « on » in the 100–1700 m/z range by synchronizing quadrupole switching events with the precursor elution profile from the TIMS device. The cycle duty time was set to 100%, accommodating as many MS/MS in the PASEF frame as possible. Singly charged precursor ions were excluded from the TIMS stage by tuning the TIMS using the otof control software, (Bruker Daltonik GmbH). Precursors for MS/MS were picked from an intensity threshold of 2500 arbitrary units (a.u.), refragmented and summed until reaching a ‘target value’ of 20.000 a.u. taking into account a dynamic exclusion of 0.40 s elution gap.

Samples from each of the three treatments (DMSO control, 5 and 100 µg/L DBP) were analyzed to identify and quantify the SHs by LC-MS/MS. Proteins of interest (Table 1) were identified with high confidence based on the following criteria:

- (1) Absent in no probe (DMSO) samples, (2) in labelled samples with probe (dB-FP), presence of unique peptides in at least three out of four replicates with at least two of four of the replicates showing LFQ ratios.
- When proteins are present in no-probe samples, (3) a significant difference *p* < 0.05 (*t*-test, 2-tailed distribution) * between labelled samples with probe (dB-FP) and no-probe (DMSO) samples, (4) an abundance of at least two times higher in labelled samples with probe than in no-probe (DMSO) samples.

* Log2 LFQ were used for comparison with *t*-test.

Table S2 shows the selection of proteins displayed in Table 1 with the LFQ values, *p*-values and ratio corresponding to each protein.

Protein quantification and data analysis. The mass spectrometry data were analyzed using Maxquant version 1.6.17 [65]. The database used was a concatenation of *Danio rerio* sequences from the Uniprot databases (release 2021-04) and a list of contaminant sequences from Maxquant. The enzyme specificity was trypsin’s. The precursor’s and fragment’s mass tolerances were set to 20 ppm. Carbamidomethylation of cysteins was set as a permanent modification, and acetylation of protein N-terminus and oxidation of methionines were set as variable modifications. A second peptide search was allowed and minimal length of peptides was set at seven amino acids. False discovery rate (FDR) was kept below 1% on both peptides and proteins. Label-free protein quantification (LFQ) was performed using both unique and razor peptides. At least two peptide ratios present in two distinct replicas are necessary to provide an LFQ intensity. The “match between runs” (MBR) option was not allowed and the normalization was skipped. For differential analysis, LFQ results from MaxQuant were imported into the Perseus software (version 1.6.15) [66]. Reverse and contaminant proteins were excluded from analysis. The data were transformed to log2. The number of Valid Value per condition (T, C5, C100) was calculated.

Statistical analyses. All data were analyzed using the *t*-test to determine statistical differences between experimental groups. The *p*-values < 0.05 were considered significant for all statistical analyses except for RT-qPCR data, where *p* < 0.01 was considered significant.

## Figures and Tables

**Table 1:** *Danio rerio* proteins enriched by dB-FP affinity purification. For each protein identified we have the following information: name of the gene, name of the protein, Uniprot accession, mass (kDa), fraction in which it was identified (S for soluble and I for insoluble), type of SH (SP for serine protease and mSH for metabolic serine hydrolase), unique peptides identified in each of the 4 biological replicates and in each sample (100 µg/L DBP, 5 µg/L DBP and control). For each sample we have the unique peptides identified without probe (No probe) and those identified in the presence of probe (dB-FP).

Gene	Protein Name	Uniprot Accession	Mass (kDa)	S/I	SP/mSH	Unique Peptides					
						100 µg/L DBP		5 µg/L DBP		Control (DMSO)	
						No probe	dB-FP	No probe	dB-FP	No probe	dB-FP
*aadac*	Arylacetamide deacetylase	E7F2W1_DANRE	46.923	S	mSH	0, 0, 0, 0	9, 6, 7, 12	0, 0, 0, 0	8, 7, 8, 13	0, 0, 0, 0	9, 7, 9, 13
*abhd10a*	Abhydrolase domain-containing 10, depalmitoylase a	A0A0R4ILM1_DANRE	31.639	S	mSH	0, 0, 0, 0	8, 3, 6, 9	0, 0, 0, 0	6, 8, 8, 11	0, 0, 0, 0	6, 3, 6, 8
*abhd10b*	Transgelin 3b	E7F9Y7_DANRE	31.94	S	mSH	0, 0, 0, 0	11, 8, 9, 11	0, 0, 0, 0	12, 11, 12, 12	0, 0, 0, 0	12, 8, 11, 11
*abhd17ab*	Abhydrolase domain-containing 17A, depalmitoylase b	A0A2R8PY04_DANRE	32.846	S	SP	0, 0, 0, 0	4, 1, 3, 5	0, 0, 0, 0	6, 5, 4, 5	0, 0, 0, 0	6, 1, 5, 5
*abhd17b*	Abhydrolase domain-containing 17B, depalmitoylase (Fragment)	A3KPQ6_DANRE	32.28	S	SP	0, 0, 0, 0	4, 4, 5, 6	0, 0, 0, 0	5, 5, 5, 8	0, 0, 0, 0	5, 4, 4, 7
*abhd6a*	Abhydrolase domain-containing 6, acylglycerol lipase a	E7F881_DANRE	38.103	S	mSH	0, 0, 0, 0	5, 3, 5, 9	0, 0, 0, 0	5, 9, 4, 9	0, 0, 0, 0	7, 3, 4, 7
*acot14*	Acyl-CoA thioesterase 14	Q5RHG4_DANRE	49.161	S	mSH	0, 0, 0, 0	4, 1, 3, 4	0, 0, 0, 0	4, 4, 4, 6	0, 0, 0, 0	3, 2, 3, 5
*acot18*	Acyl-CoA thioesterase 18 (Fragment)	Q5RH36_DANRE	48.765	S	mSH	0, 0, 0, 0	5, 5, 5, 8	0, 0, 0, 0	4, 7, 6, 11	0, 0, 0, 0	6, 6, 4, 9
*acot20*	Acyl-CoA thioesterase 20	Q5SPG8_DANRE	48.601	S	mSH	0, 0, 0, 0	10, 4, 10, 15	0, 0, 0, 0	9, 9, 13, 16	0, 0, 0, 0	8, 3, 8, 12
*afmid*	Kynurenine formamidase (Fragment)	A0A0R4INQ3_DANRE	30.694	S	mSH	0, 0, 0, 0	6, 2, 7, 5	0, 0, 0, 0	4, 6, 9, 6	0, 0, 0, 0	5, 5, 7, 6
*apeh*	Acyl-peptide hydrolase	F1QTY6_DANRE	83.262	S	SP	0, 0, 0, 0	6, 3, 3, 6	0, 0, 0, 0	5, 6, 9, 9	0, 0, 0, 0	5, 3, 10, 7
*ccdc57*	3-hydroxyacyl-[acyl-carrier-protein] dehydratase	E7F5V3_DANRE	274.19	S	mSH	0, 0, 0, 0	23, 11, 21, 40	0, 0, 0, 0	21, 21, 26, 26	0, 0, 0, 0	25, 9, 28, 28
				I	mSH	0, 0, 0, 0	7, 7, 5, 4	0, 0, 0, 0	6, 7, 10, 3	0, 0, 0, 0	7, 5, 6, 3
*cel.1*	Carboxylic ester hydrolase	F1R1T7_DANRE	60.783	S	mSH	0, 0, 0, 0	7, 5, 5, 6	0, 0, 0, 0	3, 5, 6, 7	0, 0, 0, 0	3, 5, 4, 7
*cel.2*	Carboxylic ester hydrolase	F6P131_DANRE	60.555	S	mSH	0, 0, 0, 0	5, 5, 4, 8	0, 0, 0, 0	4, 5, 6, 10	0, 0, 0, 0	2, 4, 6, 8
*cela1.6*	Chymotrypsin-like elastase family member 1, tandem duplicate 6	A0A2R8RMN0_DANRE	29.475	S	SP	0, 4, 1, 3	5, 7, 3, 3	0, 1, 1, 3	2, 7, 3, 5	0, 4, 1, 3	2, 7, 1, 5
*ces2a*	Carboxylic ester hydrolase	Q1LYL6_DANRE	60.611	S	mSH	0, 0, 0, 0	7, 8, 8, 7	0, 0, 0, 0	8, 10, 9, 7	0, 0, 0, 0	8, 7, 8, 7
*ces2a*	Carboxylic ester hydrolase (Fragment)	Q6GMJ1_DANRE	60.634	S	mSH	0, 0, 0, 0	4, 5, 7, 5	0, 0, 0, 0	6, 5, 8, 4	0, 0, 0, 0	7, 4, 5, 5
*ces2b*	Carboxylic ester hydrolase	A0A0R4IMR1_DANRE	29.286	S	mSH	0, 0, 0, 0	4, 5, 5, 3	0, 0, 0, 0	4, 4, 4, 3	0, 0, 0, 0	4, 4, 4, 3
*ces2b*	Carboxylesterase 2b (Fragment)	A0A0R4IU19_DANRE	104.95	S	mSH	0, 0, 0, 0	21, 23, 17, 16	0, 0, 0, 0	20, 25, 20, 19	0, 0, 0, 0	19, 18, 20, 20
			104.95	I	mSH	0, 0, 0, 0	4, 7, 3, 4	0, 0, 0, 0	1, 3, 7, 3	0, 0, 0, 0	5, 5, 3, 2
*ces3*	Carboxylic ester hydrolase	A0A2R8QSI8_DANRE	59.431	S	mSH	0, 0, 0, 0	2, 2, 2, 2	0, 0, 0, 0	2, 2, 2, 2	0, 0, 0, 0	1, 2, 2, 2
*ces3*	Carboxylic ester hydrolase	Q1LUZ9_DANRE	60.297	S	mSH	0, 0, 0, 0	4, 3, 4, 4	0, 0, 0, 0	4, 3, 4, 4	0, 0, 0, 0	3, 2, 4, 4
*cpvl*	Carboxypeptidase vitellogenic-like	Q7ZU43_DANRE	54.514	S	SP	0, 0, 0, 0	13, 11, 12, 15	0, 0, 0, 0	15, 13, 15, 13	0, 0, 0, 0	13, 10, 13, 12
*ctrb1*	Chymotrypsinogen B1	F1QFX9_DANRE	28.245	S	SP	0, 0, 0, 0	1, 5, 1, 1	0, 0, 0, 0	0, 4, 2, 1	0, 0, 0, 0	0, 5, 0, 2
*Ctsa*	Carboxypeptidase	A0A0R4ILY1_DANRE	53.007	S	SP	0, 0, 1, 1	8, 5, 7, 6	0, 0, 0, 1	6, 7, 8, 6	0, 0, 0, 0	8, 5, 6, 5
				I	SP	0, 0, 0, 0	4, 4, 3, 3	0, 0, 0, 0	3, 1, 2, 3	0, 0, 0, 1	3, 1, 3, 1
*dpp4*	Dipeptidyl peptidase 4	B5DDZ4_DANRE	84.667	S	mSH	0, 0, 0, 0	14, 12, 11, 6	0, 0, 0, 0	12, 13, 12, 10	0, 0, 0, 0	12, 10, 12, 12
*dpp9*	Dipeptidyl-peptidase 9	A0A2R8QM73_DANRE	98.325	S	SP	0, 0, 0, 0	18, 8, 13, 7	0, 0, 0, 0	17, 13, 18, 11	0, 0, 0, 1	16, 7, 17, 13
				I	SP	0, 0, 0, 0	5, 6, 6, 4	0, 0, 0, 0	6, 4, 9, 6	0, 0, 0, 0	7, 6, 4, 5
*ela2*	Elastase 2	A0A0R4IXD6_DANRE	28.887	S	SP	0, 2, 0, 0	3, 5, 1, 2	0, 1, 0, 0	0, 5, 2, 2	0, 0, 0, 1	0, 4, 1, 3
*esd*	S-formylglutathione hydrolase	Q567K2_DANRE	31.171	S	SP	0, 0, 0, 0	6, 4, 5, 6	0, 0, 0, 0	5, 6, 8, 6	0, 0, 0, 1	3, 3, 3, 7
*faah*	Fatty acid amide hydrolase	F1RCW3_DANRE	65.153	S	SP	0, 0, 0, 0	1, 1, 3, 11	0, 0, 0, 0	2, 6, 4, 6	0, 0, 0, 0	2, 0, 6, 7
*faah2b*	Fatty-acid amide hydrolase 2-B	F1QM44_DANRE	57.151	S	SP	0, 0, 0, 0	12, 8, 8, 15	0, 0, 0, 0	12, 11, 13, 13	0, 0, 0, 0	14, 7, 13, 17
*fap*	Dipeptidyl peptidase 4	B0R1C4_DANRE	85.806	S	SP	0, 0, 0, 0	25, 28, 27, 23	0, 0, 0, 0	28, 34, 30, 28	0, 0, 0, 0	29, 25, 26, 29
				I	SP	0, 0, 0, 0	9, 14, 15, 16	0, 0, 0, 0	12, 12, 14, 16	0, 0, 0, 0	10, 16, 15, 7
*Lcat*	Lecithin-cholesterol acyltransferase	A0A0R4IDL2_DANRE	49.139	S	mSH	0, 0, 0, 0	8, 6, 10, 10	0, 0, 0, 0	7, 10, 10, 10	0, 0, 0, 0	8, 5, 9, 9
*lypla1*	Lysophospholipase 1	Q568J5_DANRE	21.218	S	mSH	0, 0, 0, 0	2, 1, 1, 4	0, 0, 0, 0	1, 3, 1, 4	0, 0, 0, 0	1, 2, 1, 4
*lypla2*	Lysophospholipase 2	Q6PBW8_DANRE	25.067	S	mSH	0, 0, 0, 0	8, 8, 11, 10	0, 0, 0, 0	11, 11, 10, 10	0, 0, 0, 0	10, 10, 10, 10
				I	mSH	0, 0, 0, 0	2, 2, 1, 1	0, 0, 0, 0	0, 1, 1, 0	0, 0, 0, 0	2, 0, 2, 2
*mgll*	Monoglyceride lipase	Q7ZWC2_DANRE	33.66	S	mSH	0, 0, 0, 0	7, 4, 7, 10	0, 0, 0, 0	8, 9, 9, 11	0, 0, 0, 0	6, 7, 6, 10
*N/A*	2-arachidonoylglycerol hydrolase ABHD12	A0A2R8QQD0_DANRE	40.472	S	SP	0, 0, 0, 0	4, 4, 4, 4	0, 0, 0, 0	4, 5, 5, 4	0, 0, 0, 0	5, 4, 4, 5
*nceh1b.1*	Neutral cholesterol ester hydrolase 1b, tandem duplicate 1	F1Q8P9_DANRE	45.39	S	mSH	0, 0, 0, 0	7, 2, 5, 7	0, 0, 0, 0	5, 7, 8, 7	0, 0, 0, 0	3, 3, 4, 7
*pla2g15*	Phospholipase A2, group XV (Fragment)	A0A0R4IE29_DANRE	53.521	S	mSH	0, 0, 0, 0	1, 3, 5, 9	0, 0, 0, 0	2, 7, 7, 9	0, 0, 0, 0	2, 5, 4, 8
*pla2g7*	Platelet-activating factor acetylhydrolase	Q5RHM0_DANRE	50.429	S	mSH	0, 0, 0, 0	1, 2, 4, 8	0, 0, 0, 0	2, 4, 4, 10	0, 0, 0, 0	2, 2, 3, 7
*ppme1*	Protein phosphatase methylesterase 1	Q7ZV37_DANRE	41.64	S	mSH	0, 0, 0, 0	4, 0, 4, 6	0, 0, 0, 0	3, 3, 5, 9	0, 0, 0, 0	4, 1, 5, 8
*ppt2b*	Novel protein similar to verebrate palmitoyl-protein thioesterase 2 (PPT2)	A3KPZ6_DANRE	32.366	S	mSH	0, 0, 0, 0	1, 2, 1, 2	0, 0, 0, 0	2, 2, 2, 3	0, 0, 0, 0	2, 2, 1, 3
*prcp*	Prolylcarboxypeptidase (Angiotensinase c)	Q6DG46_DANRE	55.132	S	SP	0, 0, 0, 0	3, 0, 0, 5	0, 0, 0, 0	0, 2, 3, 4	0, 0, 0, 0	0, 0, 0, 5
*prep*	Prolyl endopeptidase	Q503E2_DANRE	80.336	S	SP	0, 0, 0, 1	22, 14, 20, 22	0,0,0,1	20, 20, 25, 22	0, 1, 0, 0	19, 11, 22, 25
*rbbp9*	Retinoblastoma-binding protein 9	Q1MT41_DANRE	20.887	S	mSH	0, 0, 0, 0	6, 3, 4, 4	0, 0, 0, 0	6, 6, 6, 4	0, 0, 0, 0	4, 2, 4, 5
*si:ch211-117n7.6*	Si:ch211-117n7.6	A8E7H0_DANRE	38.7	S	mSH	0, 0, 0, 0	5, 2, 6, 8	0, 0, 0, 0	7, 9, 9, 8	0, 0, 0, 0	7, 3, 5, 8
*si:ch211-117n7.7*	Si:ch211-117n7.7	A8E7G9_DANRE	39.501	S	mSH	0, 0, 0, 0	7, 5, 3, 9	0, 0, 0, 0	7, 6, 8, 9	0, 0, 0, 0	5, 5, 5, 8
*si:ch211-122f10.4*	Carboxypeptidase	F1QYP6_DANRE	51.525	S	mSH	0, 0, 0, 0	7, 7, 4, 5	0, 0, 0, 0	8, 4, 5, 6	0, 0, 0, 0	6, 7, 6, 5
*si:ch211-71n6.4*	Carboxylic ester hydrolase	A0A0R4IYT8_DANRE	67.565	S	mSH	0, 0, 0, 0	11, 7, 6, 14	0, 0, 0, 0	9, 11, 12, 15	0, 0, 0, 0	13, 3, 8, 14
*si:ch73-89b15.3*	Carboxylic ester hydrolase	A0A0R4IPW5_DANRE	63.523	S	mSH	0, 0, 0, 0	3, 1, 3, 10	0, 0, 0, 0	5, 6, 6, 10	0, 0, 0, 0	5, 1, 4, 11
*si:dkey-21e2.16*	Si:dkey-21e2.16 (Fragment)	Q1LUQ6_DANRE	25.62	S	SP	0, 0, 0, 0	2, 2, 0, 3	0, 0, 0, 0	0, 4, 2, 4	0, 0, 0, 0	0, 2, 0, 3
*siae*	Sialic acid acetylesterase	Q1LUX8_DANRE	56.624	S	SP	0, 0, 0, 0	3, 2, 2, 6	0, 0, 0, 0	2, 3, 3, 2	0, 0, 0, 0	0, 1, 2, 4
*tpp2*	Tripeptidyl-peptidase 2	R4GDQ0_DANRE	139.25	I	SP	0, 0, 0, 0	8, 8, 7, 7	0, 0, 0, 0	11, 13, 17, 10	0, 0, 0, 0	20, 6, 8, 9
*zgc:154142*	Zgc:154142	A5PKM4_DANRE	118.63	S	SP	0, 0, 0, 0	1, 1, 1, 2	0, 0, 0, 0	0, 7, 2, 4	0, 0, 0, 0	0, 3, 1, 3
Other proteins											
*clic3*	Chloride intracellular channel 3	E7F4S2_DANRE	151.36	S	-	0, 0, 0, 0	1, 0, 0, 6	0, 0, 0, 0	1, 2, 3, 6	0, 0, 0, 0	0, 0, 2, 8
*a2ml*	Alpha-2-macroglobulin-like	A0A0R4IDD1_DANRE	159.76	S	-	0, 0, 0, 0	11, 12, 9, 10	0, 0, 0, 0	5, 14, 11, 11	0, 0, 0, 0	5, 10, 4, 12
*colq*	Collagen-like tail subunit (single strand of homotrimer) of asymmetric acetylcholinesterase	F1Q7Y1_DANRE	47.825	I	-	0, 0, 3, 0	3, 5, 3, 3	0, 0, 0, 0	3, 4, 4, 3	1, 0, 1, 0	4, 3, 2, 3

**Table 2:** Primer sequences of genes tested in this study.

Genes and [Reference]	Primers (5′–3′)	Amplicon (pb)
Elongation factor 1 alpha (ef1-α) [64]	Foward: TACAAATGCGGTGGAATCGAC Reverse: GTCAGCCTGAGAAGTACCAGT	248
Ribosomal protein L 13a (rpl13a) [64]	Foward: TCTGGAGGACTGTAAGAGGTATGC Reverse: AGACGCACAATCTTGAGAGCAG	149
Vitellogenin (vtg7) [62]	Foward: GCCAAAAAGCTGGGTAAACA Reverse: AGTTCCGTCTGGATTGATGG	210
Acyl-coenzyme-A-oxidase 1 (acox-1)	Foward: TCCATGAGTCCCACAACAAG Reverse: CCTTTCTTCCCCTTTCTTGC	75
17β-hydroxysteroid dehydrogenase (17b-HSD 12a)	Foward: ACCAGACCAACGGCTATTTC Reverse: ATCGCAGTTTTCTCCTCCTG	141

## Data Availability

The mass spectrometry proteomics data have been deposited to the ProteomeXchange Consortium via the PRIDE [67] partner repository with the dataset identifier PXD038229.

## Supplementary Materials

The supporting information can be downloaded at: https://www.mdpi.com/article/10.3390/ijms232416060/s1.
