# Supplementary material for: First Identification of a Large Set of Serine Hydrolases by Activity-Based Protein Profiling in Dibutyl Phthalate-Exposed Zebrafish Larvae
Source: Int J Mol Sci. 2022 Dec 16;23(24):16060. doi: 10.3390/ijms232416060 (PMC9786740; doi:10.3390/ijms232416060)
Supplement: Supplementary file 1 [file ijms-23-16060-s001.zip › Table S1.pdf]

Table S1. Mortality hatching in zebrafish embryos (*Danio rerio*) in controls and DBP exposed ones, at the beginning of the exposure (D_0) and daily for the 5 days of exposure.

	D_0	$D_0 + 1$	$D_0 + 2$	$D_0 + 3$	$D_0 + 4$	$D_0 + 5$	Mortality (total)	% Mortality	% Hatching		
									72 hpf	96 hpf	120 hpf
Control	200	5	0	0	0	0	5	3%	0%	41%	90%
Control	200	3	1	0	0	0	4	2%	5%	75%	98%
Control	200	4	0	0	0	0	4	2%	1%	76%	96%
Control	200	1	2	0	0	0	3	2%	0%	70%	98%
**Control_Mean**	**200**	**3.25**	**0.75**	**0**	**0**	**0**	**4 (± 0.8)**	**2% (± 0.4)**	**1% (± 2)**	**65% (± 16)**	**95% (± 4)**
C5	200	2	1	0	0	0	3	2%	1%	69%	94%
C5	200	4	0	0	0	0	4	2%	2%	75%	98%
C5	200	3	1	0	0	0	4	2%	2%	63%	96%
C5	200	2	2	0	0	0	4	2%	0%	61%	97%
**C5_Mean**	**200**	**2.75**	**1**	**0**	**0**	**0**	**3.75 (± 0.5)**	**2% (± 0.3)**	**1% (± 1)**	**67% (± 6)**	**96% (± 2)**
C100	200	1	1	0	0	0	2	1%	0%	88%	97%
C100	200	8	2	0	0	0	10	5%	2%	71%	95%
C100	200	3	1	2	0	0	6	3%	5%	77%	97%
C100	200	1	0	0	0	0	1	1%	0%	69%	98%
**C100_Mean**	**200**	**3.25**	**1**	**0.5**	**0**	**0**	**4.75 (± 4.1)**	**2% (± 2.1)**	**2% (± 2)**	**76% (± 9)**	**96% (± 1)**

Note: D_0 = Day 0
