# Supplementary material for: First Identification of a Large Set of Serine Hydrolases by Activity-Based Protein Profiling in Dibutyl Phthalate-Exposed Zebrafish Larvae
Source: Int J Mol Sci. 2022 Dec 16;23(24):16060. doi: 10.3390/ijms232416060 (PMC9786740; doi:10.3390/ijms232416060)
Supplement: Supplementary file 1 [file ijms-23-16060-s001.zip › Table S2.pdf]

Table S2 : Label free quantification (LFQ) of identified SHs. Quantification data were transformed to log2. Student's *t*-test was used to determine statistical differences between experimental groups. Significance was established at $p < 0.05$ in all cases. Significant Student's t-test is colored in green.

			Control								C5 (5 µg/L DBP)								C100 (100 µg/L DBP)																				
			No probe				With probe				No probe				With probe				No probe				With probe															
T: Protein IDs	T: Fasta headers	Fraction	log 2 LFQ intensity C_1	log 2 LFQ intensity C_2	log 2 LFQ intensity C_3	log 2 LFQ intensity C_4	log 2 LFQ intensity CP_1	log 2 LFQ intensity CP_2	log 2 LFQ intensity CP_3	log 2 LFQ intensity CP_4	log 2 LFQ intensity C5_1	log 2 LFQ intensity C5_2	log 2 LFQ intensity C5_3	log 2 LFQ intensity C5_4	log 2 LFQ intensity C5P_1	log 2 LFQ intensity C5P_2	log 2 LFQ intensity C5P_3	log 2 LFQ intensity C5P_4	log 2 LFQ intensity C100_1	log 2 LFQ intensity C100_2	log 2 LFQ intensity C100_3	log 2 LFQ intensity C100_4	log 2 LFQ intensity C100P_1	log 2 LFQ intensity C100P_2	log 2 LFQ intensity C100P_3	log 2 LFQ intensity C100P_4	Student's *t*-test Significant C5P_CP	Student's *t*-test p-value C5P_CP	Log2 Ratio C5P_CP	Student's *t*-test Significant C100P_CP	Student's *t*-test p-value C100P_CP	Log2 Ratio C100P_CP	Valid values C	Valid values CP	Valid values C5	Valid values C5P	Valid values C100	Valid values C100P
A0A0R4IDL2 DANRE Lecithir	tr|A0A0R4IDL2|A0A0R4IDL2 DANRE Lecithin-	S	NaN	NaN	NaN	NaN	16,1068	16,0751	16,4136	18,0634	NaN	NaN	NaN	NaN	16,0407	16,613	17,3353	18,5968	NaN	NaN	NaN	NaN	16,313	15,2798	17,0162	18,1879		0,531637	0,48172		0,965847	0,0344751	0	4	0	4	0	4
A0A0R4IE29 DANRE Phosph	tr|A0A0R4IE29|A0A0R4IE29 DANRE Phospho	S	NaN	NaN	NaN	NaN	12,9289	15,8827	15,6512	18,0903	NaN	NaN	NaN	NaN	15,8909	16,9348	16,7155	18,4683	NaN	NaN	NaN	NaN	NaN	14,6186	15,6204	18,6244		0,293922	1,36406		0,702591	0,649522	0	4	0	4	0	3
A0A0R4ILM1 DANRE Abhydı	tr|A0A0R4ILM1|A0A0R4ILM1 DANRE Abhydr	S	NaN	NaN	NaN	NaN	14,405	13,3625	14,7138	16,5862	NaN	NaN	NaN	NaN	14,9178	15,1478	15,7481	16,8746	NaN	NaN	NaN	NaN	14,1885	13,2751	15,3215	16,5067		0,301932	0,905201		0,955787	0,0560808	0	4	0	4	0	4
A0A0R4ILY1 DANRE Carboxy	tr|A0A0R4ILY1|A0A0R4ILY1 DANRE Carboxyp	S	NaN	NaN	NaN	NaN	19,9635	19,5549	19,3899	19,7121	NaN	NaN	NaN	NaN	19,2014	20,3152	21,1616	21,3621	NaN	NaN	NaN	NaN	20,1254	17,7246	20,4474	21,7836		0,142456	0,854952		0,683881	0,365142	0	4	0	4	0	4
A0A0R4ILY1 DANRE Carboxy	tr|A0A0R4ILY1|A0A0R4ILY1 DANRE Carboxyp	I	NaN	NaN	NaN	NaN	14,2999	NaN	15,1324	NaN	NaN	NaN	NaN	NaN	12,9003	NaN	17,0572	14,9239	NaN	NaN	NaN	NaN	15,9577	16,3251	15,504	16,1138		0,886929	0,244335	+	0,0260643	1,25904	0	2	0	3	0	4
A0A0R4IMR1 DANRE Carbo	tr|A0A0R4IMR1|A0A0R4IMR1 DANRE Carbox	S	NaN	NaN	NaN	NaN	17,0387	17,5265	16,9556	17,9274	NaN	NaN	NaN	NaN	16,6354	17,9405	18,6346	17,4571	NaN	NaN	NaN	NaN	17,8656	18,1119	17,7223	17,1674		0,546767	0,304843		0,285044	0,354746	0	4	0	4	0	4
Q568J5 DANRE Lypla1 prote	tr|Q568J5|Q568J5 DANRE Lypla1 protein OS	S	NaN	NaN	NaN	NaN	NaN	13,3622	13,5468	15,5112	NaN	NaN	NaN	NaN	13,326	14,1836	14,185	15,7175	NaN	NaN	NaN	NaN	13,2438	12,6363	13,6203	15,5788		0,806198	0,212981		0,71204	-0,37025	0	3	0	4	0	4
A0A0R4INQ3 DANRE Kynure	tr|A0A0R4INQ3|A0A0R4INQ3 DANRE Kynure	S	NaN	NaN	NaN	NaN	15,4547	16,0033	16,9921	16,7917	NaN	NaN	NaN	NaN	15,5744	16,4924	16,9676	17,3046	NaN	NaN	NaN	NaN	16,076	15,6474	16,3114	16,4933		0,615355	0,27425		0,671435	-0,178454	0	4	0	4	0	4
A0A0R4IPW5 DANRE Carbo	tr|A0A0R4IPW5|A0A0R4IPW5 DANRE Carbox	S	NaN	NaN	NaN	NaN	14,9831	NaN	14,5288	16,3137	NaN	NaN	NaN	NaN	15,2826	15,6965	15,3742	16,2977	NaN	NaN	NaN	NaN	14,4166	NaN	14,7562	15,9774		0,49273	0,387569		0,768683	-0,225094	0	3	0	4	0	3
A3KPZ6 DANRE Novel prote	tr|A3KPZ6|A3KPZ6 DANRE Novel protein sim	S	NaN	NaN	NaN	NaN	14,7027	15,2098	NaN	16,1049	NaN	NaN	NaN	NaN	14,751	15,4432	15,3486	16,3477	NaN	NaN	NaN	NaN	NaN	14,3597	14,4112	16,1427		0,807367	0,133482		0,634007	-0,367937	0	3	0	4	0	3
A0A0R4IU19 DANRE Carbox	tr|A0A0R4IU19|A0A0R4IU19 DANRE Carboxy	S	NaN	NaN	NaN	NaN	18,3564	18,9319	18,8396	18,9812	NaN	NaN	NaN	NaN	18,3856	19,2036	19,3611	18,6279	NaN	NaN	NaN	NaN	19,4041	19,4575	18,36	18,5862		0,681657	0,117311		0,599235	0,174685	0	4	0	4	0	4
A0A0R4IU19 DANRE Carbox	tr|A0A0R4IU19|A0A0R4IU19 DANRE Carboxy	I	NaN	NaN	NaN	NaN	11,9637	13,2112	11,5335	11,0146	NaN	NaN	NaN	NaN	NaN	10,9893	13,2554	11,7395	NaN	NaN	NaN	NaN	12,7975	13,8148	12,5374	12,4393		0,938321	0,0639858		0,137916	0,966505	0	4	0	3	0	4
A0A0R4IXD6 DANRE Elastas	tr|A0A0R4IXD6|A0A0R4IXD6 DANRE Elastase	S	NaN	NaN	NaN	NaN	NaN	18,6944	NaN	15,9993	NaN	NaN	NaN	NaN	NaN	16,8228	13,9814	15,6362	NaN	11,9935	NaN	NaN	15,0848	18,121	NaN	14,5539		0,291794	-1,86672		0,47484	-1,42693	0	2	0	3	1	3
A0A0R4IYT8 DANRE Carboxy	tr|A0A0R4IYT8|A0A0R4IYT8 DANRE Carboxyl	S	NaN	NaN	NaN	NaN	15,7266	14,5305	16,3498	17,5516	NaN	NaN	NaN	NaN	15,7845	16,2318	16,9104	17,5921	NaN	NaN	NaN	NaN	15,5814	14,6104	16,2592	17,9768		0,457718	0,590043		0,94572	0,0673158	0	4	0	4	0	4
Q5RHG4 DANRE Acyl-CoA th	tr|Q5RHG4|Q5RHG4 DANRE Acyl-CoA thioest	S	NaN	NaN	NaN	NaN	14,4079	13,6472	14,7345	16,5053	NaN	NaN	NaN	NaN	14,0684	14,8778	15,1894	16,9188	NaN	NaN	NaN	NaN	14,4093	13,3176	14,33	16,8142		0,624232	0,43985		0,915429	-0,106001	0	4	0	4	0	4
A0A2R8PY04 DANRE Abhydı	tr|A0A2R8PY04|A0A2R8PY04 DANRE Abhydr	S	NaN	NaN	NaN	NaN	16,0616	NaN	16	16,1061	NaN	NaN	NaN	NaN	15,833	14,4805	15,6925	16,2555	NaN	NaN	NaN	NaN	15,1173	NaN	14,5835	16,4405		0,326783	-0,490526		0,288826	-0,675501	0	3	0	4	0	3
E7F9Y7 DANRE Transgelin 3l	tr|E7F9Y7|E7F9Y7 DANRE Transgelin 3b OS=l	S	NaN	NaN	NaN	NaN	17,2938	16,3665	17,4337	18,9754	NaN	NaN	NaN	NaN	17,1296	17,6448	18,5149	19,1884	NaN	NaN	NaN	NaN	16,9297	16,0208	17,9882	19,5328		0,427619	0,602056		0,917277	0,100523	0	4	0	4	0	4
A0A2R8QM73 DANRE Dipep	tr|A0A2R8QM73|A0A2R8QM73 DANRE Dipe	S	NaN	NaN	NaN	NaN	16,0374	15,215	16,7844	15,6047	NaN	NaN	NaN	NaN	16,0975	16,1277	16,566	15,4212	NaN	NaN	NaN	NaN	16,4426	14,9326	15,8522	14,5123		0,740253	0,142721		0,421752	-0,475455	0	4	0	4	0	4
A0A2R8QM73 DANRE Dipep	tr|A0A2R8QM73|A0A2R8QM73 DANRE Dipe	I	NaN	NaN	NaN	NaN	13,1987	13,2208	12,6999	12,8794	NaN	NaN	NaN	NaN	13,1476	12,6756	13,8825	12,6187	NaN	NaN	NaN	NaN	12,0754	13,0975	12,8701	12,8644		0,806854	0,0814035		0,329673	-0,272825	0	4	0	4	0	4
A0A2R8QQD0 DANRE 2-arac	tr|A0A2R8QQD0|A0A2R8QQD0 DANRE 2-ara	S	NaN	NaN	NaN	NaN	15,5128	15,8135	16,2344	17,8673	NaN	NaN	NaN	NaN	15,81	16,7465	17,3806	17,9727	NaN	NaN	NaN	NaN	15,665	15,118	16,3046	18,4104		0,409278	0,620471		0,98494	0,01754	0	4	0	4	0	4
A0A2R8QSI8 DANRE Carbox	tr|A0A2R8QSI8|A0A2R8QSI8 DANRE Carboxy	S	NaN	NaN	NaN	NaN	NaN	14,1185	14,4037	16,2425	NaN	NaN	NaN	NaN	13,4299	15,5767	15,9583	16,2061	NaN	NaN	NaN	NaN	14,5765	14,3857	13,6092	16,2042		0,70761	0,371198		0,800009	-0,227669	0	3	0	4	0	4
B5DDZ4 DANRE Dipeptidyl p	tr|B5DDZ4|B5DDZ4 DANRE Dipeptidyl peptic	S	NaN	NaN	NaN	NaN	15,027	15,9043	15,62	15,7253	NaN	NaN	NaN	NaN	15,2868	16,0001	16,1928	16,0826	NaN	NaN	NaN	NaN	15,3715	15,0223	15,012	15,3572		0,294054	0,321396		0,128627	-0,378448	0	4	0	4	0	4
A0A2R8RMN0 DANRE Chym	tr|A0A2R8RMN0|A0A2R8RMN0 DANRE Chyr	S	NaN	15,0704	NaN	14,4117	13,5969	19,9065	NaN	17,2064	NaN	NaN	NaN	14,2341	13,6109	18,9941	15,5755	16,9179	NaN	13,7659	NaN	13,8091	15,1871	19,5399	15,6448	16,5915		0,769659	-0,628639		0,935778	-0,162435	2	3	1	4	2	4
A3KPQ6 DANRE Abhydrolas	tr|A3KPQ6|A3KPQ6 DANRE Abhydrolase don	S	NaN	NaN	NaN	NaN	15,7458	15,5929	15,8046	17,2032	NaN	NaN	NaN	NaN	15,3904	16,761	16,6925	17,2616	NaN	NaN	NaN	NaN	14,9425	14,9199	15,6045	17,3067		0,452666	0,439744		0,581112	-0,393242	0	4	0	4	0	4
A8E7G9 DANRE Si:ch211-11	tr|A8E7G9|A8E7G9 DANRE Si:ch211-117n7.7	S	NaN	NaN	NaN	NaN	15,1266	15,0015	14,9052	17,0226	NaN	NaN	NaN	NaN	15,0821	16,1686	16,0369	17,4808	NaN	NaN	NaN	NaN	15,1588	14,547	15,3652	17,3956		0,373731	0,678102		0,901854	0,10264	0	4	0	4	0	4
A8E7H0 DANRE Si:ch211-11	tr|A8E7H0|A8E7H0 DANRE Si:ch211-117n7.6	S	NaN	NaN	NaN	NaN	16,6865	16,6675	17,1939	18,9175	NaN	NaN	NaN	NaN	16,4096	17,3648	17,7579	19,2028	NaN	NaN	NaN	NaN	16,4551	15,7805	17,7356	19,2951		0,700526	0,31743		0,959446	-0,0497694	0	4	0	4	0	4
Q5RH36 DANRE Acyl-CoA th	tr|Q5RH36|Q5RH36 DANRE Acyl-CoA thioest	S	NaN	NaN	NaN	NaN	14,4967	14,5083	14,5051	15,7688	NaN	NaN	NaN	NaN	14,0738	16,006	15,0666	16,8418	NaN	NaN	NaN	NaN	14,4457	13,8873	13,807	16,2187		0,354874	0,677318		0,733251	-0,230072	0	4	0	4	0	4
B0R1C4 DANRE Dipeptidyl p	tr|B0R1C4|B0R1C4 DANRE Dipeptidyl peptid	S	NaN	NaN	NaN	NaN	17,7182	17,1974	17,5391	18,4504	NaN	NaN	NaN	NaN	17,7146	18,3847	18,2759	18,614	NaN	NaN	NaN	NaN	17,2918	16,8308	17,2798	17,8693		0,16133	0,521038		0,274262	-0,408355	0	4	0	4	0	4
B0R1C4 DANRE Dipeptidyl p	tr|B0R1C4|B0R1C4 DANRE Dipeptidyl peptid	I	NaN	NaN	NaN	NaN	13,3442	15,5747	15,2104	13,2829	NaN	NaN	NaN	NaN	14,3593	14,3724	14,9296	14,2741	NaN	NaN	NaN	NaN	13,9605	15,2493	15,0866	15,4875		0,840738	0,130783		0,425207	0,592932	0	4	0	4	0	4
E7F2W1 DANRE Arylacetam	tr|E7F2W1|E7F2W1 DANRE Arylacetamide d	S	NaN	NaN	NaN	NaN	16,0298	14,7826	16,3903	17,8564	NaN	NaN	NaN	NaN	15,6753	16,6544	16,8676	17,9204	NaN	NaN	NaN	NaN	15,3434	14,4764	16,1191	18,0599		0,53506	0,514659		0,798292	-0,265045	0	4	0	4	0	4
E7F5V3 DANRE 3-hydroxyac	tr|E7F5V3|E7F5V3 DANRE 3-hydroxyacyl-[ac	S	NaN	NaN	NaN	NaN	16,6317	16,3714	17,5386	17,8525	NaN	NaN	NaN	NaN	16,5907	17,2793	17,8697	17,8858	NaN	NaN	NaN	NaN	16,4086	15,7962	17,3846	18,2789		0,535549	0,307858		0,846722	-0,131405	0	4	0	4	0	4
E7F5V3 DANRE 3-hydroxyac	tr|E7F5V3|E7F5V3 DANRE 3-hydroxyacyl-[ac	I	NaN	NaN	NaN	NaN	12,7044	13,224	13,5925	12,5223	NaN	NaN	NaN	NaN	13,6917	13,3304	14,2456	12,2472	NaN	NaN	NaN	NaN	12,745	13,6118	12,9753	13,1661		0,478711	0,367931		0,722684	0,11374	0	4	0	4	0	4
E7F881 DANRE Abhydrolase	tr|E7F881|E7F881 DANRE Abhydrolase doma	S	NaN	NaN	NaN	NaN	14,9216	15,2862	15,0119	16,745	NaN	NaN	NaN	NaN	15,2301	16,223	15,5017	17,0756	NaN	NaN	NaN	NaN	15,4595	15,2538	15,1808	16,9584		0,417106	0,51641		0,722893	0,221928	0	4	0	4	0	4
F1Q8P9 DANRE Neutral cho	tr|F1Q8P9|F1Q8P9 DANRE Neutral cholester	S	NaN	NaN	NaN	NaN	14,4617	13,3195	13,7879	16,0905	NaN	NaN	NaN	NaN	13,8957	14,6893	15,0093	15,8251	NaN	NaN	NaN	NaN	14,2376	12,5525	13,9531	16,9725		0,566457	0,439933		0,990288	0,0140219	0	4	0	4	0	4
F1QM44 DANRE Fatty-acid a	tr|F1QM44|F1QM44 DANRE Fatty-acid amid	S	NaN	NaN	NaN	NaN	16,2709	14,5164	16,745	18,0275	NaN	NaN	NaN	NaN	16,2853	16,7213	17,3513	18,286	NaN	NaN	NaN	NaN	16,0801	14,6833	16,3779	18,7134		0,397369	0,771014		0,949093	0,0737143	0	4	0	4	0	4
F1QTY6 DANRE Acyl-peptide	tr|F1QTY6|F1QTY6 DANRE Acyl-peptide hydr	S	NaN	NaN	NaN	NaN	13,8183	13,5454	15,9034	15,8014	NaN	NaN	NaN	NaN	13,8097	15,0883	16,3565	15,6816	NaN	NaN	NaN	NaN	14,5994	13,4926	14,5444	15,7804		0,594075	0,466893		0,842275	-0,162903	0	4	0	4	0	4
F1QYP6 DANRE Carboxypep	tr|F1QYP6|F1QYP6 DANRE Carboxypeptidase	S	NaN	NaN	NaN	NaN	17,4847	17,0073	18,1736	18,2027	NaN	NaN	NaN	NaN	17,6604	18,0874	17,9947	18,2104	NaN	NaN	NaN	NaN	18,2604	17,159	15,6601	18,2674		0,418341	0,271135		0,596625	-0,38039	0	4	0	4	0	4
F1R1T7 DANRE Carboxylic e	tr|F1R1T7|F1R1T7 DANRE Carboxylic ester hy	S	NaN	NaN	NaN	NaN	15,1954	15,0068	14,3404	16,8969	NaN	NaN	NaN	NaN	14,9349	15,8187	16,2205	16,5475	NaN	NaN	NaN	NaN	15,7568	14,6473	15,1311	17,0908		0,451272	0,520553		0,709455	0,296654	0	4	0	4	0	4
F1RCW3 DANRE Fatty acid a	tr|F1RCW3|F1RCW3 DANRE Fatty acid amide	S	NaN	NaN	NaN	NaN	12,1672	NaN	12,9255	15,2885	NaN	NaN	NaN	NaN	11,636	13,3915	14,0836	14,8902	NaN	NaN	NaN	NaN	NaN	NaN	12,5539	16,1171		0,973315	0,0399352		0,659943	0,87512	0	3	0	4	0	2
F6P131 DANRE Carboxylic e	tr|F6P131|F6P131 DANRE Carboxylic ester h	S	NaN	NaN	NaN	NaN	15,6222	15,9137	16,7878	18,1485	NaN	NaN	NaN	NaN	15,0086	16,8838	17,5991	17,9861	NaN	NaN	NaN	NaN	16,8266	15,6269	17,0773	18,3757		0,782562	0,251382		0,669497	0,358628	0	4	0	4	0	4
Q1LUX8 DANRE Sialic acid a	tr|Q1LUX8|Q1LUX8 DANRE Sialic acid acetyle	S	NaN	NaN	NaN	NaN	NaN	NaN	16,5733	16,0401	NaN	NaN	NaN	NaN	13,8064	15,4384	16,075	16,1436	NaN	NaN	NaN	NaN	16,1348	15,6602	16,3035	16,0602		0,321051	-0,940847		0,365285	-0,266987	0	2	0	4	0	4
Q1LUZ9 DANRE Carboxylic e	tr|Q1LUZ9|Q1LUZ9 DANRE Carboxylic ester h	S	NaN	NaN	NaN	NaN	14,6023	14,115	16,3985	17,102	NaN	NaN	NaN	NaN	15,3625	15,6609	17,2284	16,9506	NaN	NaN	NaN	NaN	15,8153	14,7504	16,2069	17,1005		0,413494	0,746185		0,648427	0,413846	0	4	0	4	0	4
Q1LYL6 DANRE Carboxylic e	tr|Q1LYL6|Q1LYL6 DANRE Carboxylic ester hy	S	NaN	NaN	NaN	NaN	18,2368	17,9676	19,3746	20,5567	NaN	NaN	9,21048	NaN	18,3804	19,2826	19,9847	20,4423	NaN	9,64286	NaN	NaN	19,1287	18,4792	19,1658	20,7127		0,535314	0,488605		0,671763	0,337704	0	4	1	4	1	4
Q1MT41 DANRE Retinoblast	tr|Q1MT41|Q1MT41 DANRE Retinoblastoma	S	NaN	NaN	NaN	NaN	14,6828	14,5304	16,7435	17,2668	NaN	NaN	NaN	NaN	15,111	16,1158	16,8503	16,726	NaN	NaN	NaN	NaN	15,5185	14,1798	16,4497	16,7913		0,641554	0,394877		0,940474	-0,0710499	0	4	0	4	0	4
Q503E2 DANRE Post-proline	tr|Q503E2|Q503E2 DANRE Post-proline cleav	S	NaN	NaN	NaN	NaN	17,28	16,3068	18,1772	18,5684	NaN	NaN	NaN	NaN	17,3197	17,565	18,5543	18,4107	NaN	NaN	NaN	NaN	17,6567	16,5101	17,1509	18,4353		0,543577	0,379316		0,830337	-0,144904	0	4	0	4	0	4
Q567K2 DANRE S-formylglu	tr|Q567K2|Q567K2 DANRE S-formylglutathic	S	NaN	NaN	NaN	NaN	15,9524	16,1629	17,809	19,5666	NaN	NaN	NaN	NaN	16,9572	17,3711	19,0217	19,4837	NaN	NaN	NaN	NaN	17,132	16,1224	17,273	18,9498		0,453299	0,835684		0,997446	-0,0034194	0	4	0	4	0	4
Q5RHM0 DANRE Platelet-ac	tr|Q5RHM0|Q5RHM0 DANRE Platelet-activat	S	NaN	NaN	NaN	NaN	11,4066	12,8682	12,9482	15,5237	NaN	NaN	NaN	NaN	11,5913	14,1862	14,2271	15,7118	NaN	NaN	NaN	NaN	NaN	12,1879	13,1374	14,6745		0,562198	0,742445		0,905963	0,146623	0	4	0	4	0	3
Q5SPG8 DANRE Acyl-CoA th	tr|Q5SPG8|Q5SPG8 DANRE Acyl-CoA thioeste	S	NaN	NaN	NaN	NaN	15,9573	14,8032	17,2665	18,6606	NaN	NaN	NaN	NaN	16,049	16,8411	18,0763	18,8812	NaN	NaN	NaN	NaN	16,3331	15,5368	16,9444	19,1113		0,477973	0,78999		0,793567	0,309506	0	4	0	4	0	4
Q6GMJ1 DANRE Carboxylic	tr|Q6GMJ1|Q6GMJ1 DANRE Carboxylic ester	S	NaN	NaN	NaN	NaN	16,7811	16,1578	18,3014	19,1162	NaN	NaN	NaN	NaN	17,2233	17,1964	18,7831	19,0745	NaN	NaN	NaN	NaN	17,7006	16,4055	18,0316	19,3803		0,589835	0,480199		0,761443	0,290373	0	4	0	4	0	4
Q6PBW8 DANRE Lysophosp	tr|Q6PBW8|Q6PBW8 DANRE Lysophospholip	S	NaN	NaN	NaN	NaN	18,605	18,4224	19,4309	20,8078	NaN	NaN	NaN	NaN	18,8386	19,5384	18,0004	20,7025	NaN	NaN	NaN	NaN	19,1274	18,1961	19,6833	20,7373		0,954888	-0,0465136		0,880179	0,119524	0	4	0	4	0	4
Q6PBW8 DANRE Lysophosp	tr|Q6PBW8|Q6PBW8 DANRE Lysophospholip	I	NaN	NaN	NaN	NaN	12,07	NaN	12,1138	13,8151	NaN	NaN	NaN	NaN	NaN	NaN	NaN	NaN	NaN	NaN	NaN	NaN	12,6024	12,7988	NaN	NaN		1	NaN		0,966193	0,0342935	0	3	0	0	0	2
Q7ZU43 DANRE Carboxypep	tr|Q7ZU43|Q7ZU43 DANRE Carboxypeptidas	S	NaN	NaN	NaN	NaN	18,6888	17,8774	18,9	19,5545	NaN	NaN	NaN	NaN	18,7193	19,0644	18,9795	19,7988	NaN	NaN	NaN	NaN	19,02	17,7998	18,784	20,1328		0,390087	0,385344		0,772126	0,179002	0	4	0	4	0	4
Q7ZV37 DANRE Protein pho	tr|Q7ZV37|Q7ZV37 DANRE Protein phosphat	S	NaN	NaN	NaN	NaN	14,8115	14,4478	15,9265	16,5184	NaN	NaN	NaN	NaN	14,2717	15,3728	16,4445	17,1646	NaN	NaN	NaN	NaN	13,906	NaN	15,5509	16,5535		0,643135	0,387365		0,92148	-0,0892214	0	4	0	4	0	3
Q7ZWC2 DANRE Monoglyce	tr|Q7ZWC2|Q7ZWC2 DANRE Monoglyceride	S	NaN	NaN	NaN	NaN	15,9772	15,5686	16,2182	18,0328	NaN	NaN	NaN	NaN	15,6825	16,7475	17,0016	18,4846	NaN	NaN	NaN	NaN	15,1725	14,9437	16,3198	18,535		0,529278	0,529858		0,840989	-0,206448	0	4	0	4	0	4
R4GDQ0 DANRE Tripeptidyl-	tr|R4GDQ0|R4GDQ0 DANRE Tripeptidyl-pept	I	NaN	NaN	NaN	NaN	15,9481	14,0424	14,1036	14,2043	NaN	NaN	NaN	NaN	15,16	15,0323	16,2314	14,2323	NaN	NaN	NaN	NaN	14,4343	14,8403	13,6177	14,422		0,375602	0,589406		0,656236	-0,24606	0	4	0	4	0	4
