# Supplementary material for: First Identification of a Large Set of Serine Hydrolases by Activity-Based Protein Profiling in Dibutyl Phthalate-Exposed Zebrafish Larvae
Source: Int J Mol Sci. 2022 Dec 16;23(24):16060. doi: 10.3390/ijms232416060 (PMC9786740; doi:10.3390/ijms232416060)
Supplement: Supplementary file 1 [file ijms-23-16060-s001.zip › Table S3.pdf]

Table S3: Gene ontology and KEGG pathway lists of the identified serine hydrolases. Gene Ontology (GO) enrichment analysis of ABPP-identified proteins were carried out using STRING database.

**Molecular Function**					
#term ID	Term description	Observed gene count	Background gene count	false discovery rate	Matching proteins (labels)
GO:0003824	Catalytic activity	48	7610	5.70e-21	cela1.6, ppme1, pla2g7, esd, acot14, cel.2, ces3, mgll, siae, acot20, abhd17ab, si:ch211-122f10.4, si:ch73-89b15.3, faah2b, cpvl, prcp, abhd6a, nceh1b.1, lcat, aadac, apeh, ces2a, si:ch211-117n7.7, ppt2b, rbbp9, ces2b, abhd12, dpp4, fap, faah, abhd10b, ctrb1, prep, ctsa, afmid, si:ch211-71n6.4, pla2g15, lypla1, dpp9, si:dkey-21e2.16, cel.1, abhd17b, lypla2, ela2, acot18, si:ch211-117n7.6, abhd10a, tpp2
GO:0016787	Hydrolase activity	47	3080	7.25e-37	cela1.6, ppme1, pla2g7, esd, acot14, cel.2, ces3, mgll, siae, acot20, abhd17ab, si:ch211-122f10.4, si:ch73-89b15.3, faah2b, cpvl, prcp, abhd6a, nceh1b.1, aadac, apeh, ces2a, si:ch211-117n7.7, ppt2b, rbbp9, ces2b, abhd12, dpp4, fap, faah, abhd10b, ctrb1, prep, ctsa, afmid, si:ch211-71n6.4, pla2g15, lypla1, dpp9, si:dkey-21e2.16, cel.1, abhd17b, lypla2, ela2, acot18, si:ch211-117n7.6, abhd10a, tpp2
GO:0016788	Hydrolase activity, acting on ester bonds	28	901	5.08e-25	ppme1, pla2g7, esd, acot14, cel.2, ces3, mgll, siae, acot20, abhd17ab, si:ch73-89b15.3, abhd6a, nceh1b.1, aadac, ces2a, si:ch211-117n7.7, ppt2b, abhd12, faah, abhd10b, pla2g15, lypla1, cel.1, abhd17b, lypla2, acot18, si:ch211-117n7.6, abhd10a
GO:0052689	Carboxylic ester hydrolase activity	23	152	2.19e-34	ppme1, pla2g7, esd, cel.2, ces3, mgll, siae, acot20, si:ch73-89b15.3, abhd6a, nceh1b.1, aadac, ces2a, si:ch211-117n7.7, abhd12, faah, abhd10b, pla2g15, lypla1, cel.1, lypla2, si:ch211-117n7.6, abhd10a
GO:0140096	Catalytic activity, acting on a protein	20	3275	0.00011	cela1.6, ppme1, abhd17ab, si:ch211-122f10.4, cpvl, prcp, apeh, ppt2b, dpp4, fap, ctrb1, prep, ctsa, lypla1, dpp9, si:dkey-21e2.16, abhd17b, lypla2, ela2, tpp2
GO:0017171	Serine hydrolase activity	15	251	6.86e-16	cela1.6, si:ch211-122f10.4, cpvl, prcp, aadac, apeh, dpp4, fap, ctrb1, prep, ctsa, dpp9, si:dkey-21e2.16, ela2, tpp2
GO:0008236	Serine-type peptidase activity	14	249	2.12e-14	cela1.6, si:ch211-122f10.4, cpvl, prcp, apeh, dpp4, fap, ctrb1, prep, ctsa, dpp9, si:dkey-21e2.16, ela2, tpp2
GO:0016298	Lipase activity	12	142	4.02e-14	cel.2, mgll, abhd6a, aadac, si:ch211-117n7.7, abhd12, faah, pla2g15, lypla1, cel.1, lypla2, si:ch211-117n7.6
**Biological Processes**					
#term ID	Term description	Observed gene count	Background gene count	false discovery rate	Matching proteins (labels)
GO:0008152	Metabolic process	45	9636	2.14e-11	cela1.6, ppme1, pla2g7, esd, acot14, cel.2, ces3, mgll, siae, acot20, abhd17ab, si:ch211-122f10.4, si:ch73-89b15.3, faah2b, cpvl, prcp, abhd6a, nceh1b.1, lcat, aadac, apeh, ces2a, si:ch211-117n7.7, ppt2b, abhd12, dpp4, fap, faah, abhd10b, ctrb1, prep, ctsa, afmid, pla2g15, lypla1, dpp9, si:dkey-21e2.16, cel.1, abhd17b, lypla2, ela2, acot18, si:ch211-117n7.6, abhd10a, tpp2
GO:0071704	Organic substance metabolic process	43	8915	6.68e-11	cela1.6, ppme1, pla2g7, esd, acot14, cel.2, ces3, mgll, siae, acot20, abhd17ab, si:ch211-122f10.4, faah2b, cpvl, prcp, abhd6a, nceh1b.1, lcat, apeh, ces2a, si:ch211-117n7.7, ppt2b, abhd12, dpp4, fap, faah, abhd10b, ctrb1, prep, ctsa, afmid, pla2g15, lypla1, dpp9, si:dkey-21e2.16, cel.1, abhd17b, lypla2, ela2, acot18, si:ch211-117n7.6, abhd10a, tpp2
GO:0044238	Primary metabolic process	41	8363	3.17e-10	cela1.6, ppme1, pla2g7, acot14, cel.2, ces3, mgll, siae, acot20, abhd17ab, si:ch211-122f10.4, faah2b, cpvl, prcp, abhd6a, nceh1b.1, lcat, apeh, ces2a, si:ch211-117n7.7, abhd12, dpp4, fap, faah, abhd10b, ctrb1, prep, ctsa, afmid, pla2g15, lypla1, dpp9, si:dkey-21e2.16, cel.1, abhd17b, lypla2, ela2, acot18, si:ch211-117n7.6, abhd10a, tpp2
GO:1901564	Organonitrogen compound metabolic process	29	6120	5.77e-05	cela1.6, ppme1, acot14, cel.2, acot20, abhd17ab, si:ch211-122f10.4, cpvl, prcp, lcat, apeh, si:ch211-117n7.7, abhd12, dpp4, fap, ctrb1, prep, ctsa, afmid, pla2g15, dpp9, si:dkey-21e2.16, cel.1, abhd17b, lypla2, ela2, acot18, si:ch211-117n7.6, tpp2
GO:0009056	Catabolic process	26	1959	1.00e-12	pla2g7, esd, cel.2, ces3, mgll, abhd17ab, si:ch211-122f10.4, si:ch73-89b15.3, faah2b, cpvl, abhd6a, nceh1b.1, aadac, ces2a, si:ch211-117n7.7, abhd12, faah, abhd10b, ctsa, afmid, pla2g15, cel.1, abhd17b, lypla2, si:ch211-117n7.6, abhd10a

GO:1901575	Organic substance catabolic process	22	1663	1.12e-10	pla2g7, esd, cel.2, mgll, abhd17ab, si:ch211-122f10.4, faah2b, cpvl, abhd6a, nceh1b.1, si:ch211-117n7.7, abhd12, faah, abhd10b, ctsa, afmid, pla2g15, cel.1, abhd17b, lypla2, si:ch211-117n7.6, abhd10a
GO:0006629	Lipid metabolic process	20	1103	2.14e-11	pla2g7, acot14, cel.2, ces3, mgll, acot20, faah2b, abhd6a, nceh1b.1, lcat, ces2a, si:ch211-117n7.7, abhd12, faah, pla2g15, lypla1, cel.1, lypla2, acot18, si:ch211-117n7.6
GO:0044248	Cellular catabolic process	18	1702	5.33e-07	esd, cel.2, mgll, si:ch211-122f10.4, si:ch73-89b15.3, cpvl, abhd6a, si:ch211-117n7.7, abhd12, faah, abhd10b, ctsa, afmid, pla2g15, cel.1, lypla2, si:ch211-117n7.6, abhd10a
GO:0044255	Cellular lipid metabolic process	16	851	1.91e-09	acot14, cel.2, mgll, acot20, abhd6a, nceh1b.1, lcat, si:ch211-117n7.7, abhd12, faah, pla2g15, lypla1, cel.1, lypla2, acot18, si:ch211-117n7.6
GO:0044281	Small molecule metabolic process	16	1733	3.23e-05	esd, acot14, cel.2, mgll, acot20, nceh1b.1, lcat, faah, abhd10b, afmid, pla2g15, lypla1, cel.1, lypla2, acot18, abhd10a
GO:0016042	Lipid catabolic process	13	272	2.14e-11	pla2g7, cel.2, mgll, faah2b, abhd6a, nceh1b.1, si:ch211-117n7.7, abhd12, faah, pla2g15, cel.1, lypla2, si:ch211-117n7.6
GO:0019752	Carboxylic acid metabolic process	11	806	0.00010	acot14, mgll, acot20, faah, abhd10b, afmid, pla2g15, lypla1, lypla2, acot18, abhd10a
GO:1901565	Organonitrogen compound catabolic process	11	1126	0.0019	abhd17ab, si:ch211-122f10.4, cpvl, si:ch211-117n7.7, abhd12, ctsa, afmid, pla2g15, abhd17b, lypla2, si:ch211-117n7.6
GO:0044242	Cellular lipid catabolic process	10	179	1.69e-09	cel.2, mgll, abhd6a, si:ch211-117n7.7, abhd12, faah, pla2g15, cel.1, lypla2, si:ch211-117n7.6
GO:0019637	Organophosphate metabolic process	10	898	0.0018	acot14, acot20, abhd6a, lcat, si:ch211-117n7.7, abhd12, afmid, pla2g15, acot18, si:ch211-117n7.6
**KEGG Pathway**					
#term ID	Term description	Observed gene count	Background gene count	false discovery rate	Matching proteins (labels)
dre00561	Glycerolipid metabolism	3	63	0.0445	cel.2, mgll, cel.1
dre00564	Glycerophospholipid metabolism	3	100	0.0428	lcat, pla2g15, lypla2
dre04142	Lysosome	4	165	0.0445	si:ch211-122f10.4, ppt2b, ctsa, pla2g15

Note: Only Molecular Function and Biological Processes with ten or more protein associations are shown.
